# Multiple, Independent T Cell Lymphomas Arising in an Experimentally FIV-Infected Cat during the Terminal Stage of Infection

**DOI:** 10.3390/v10060280

**Published:** 2018-05-24

**Authors:** Brian G. Murphy, Christina Eckstrand, Diego Castillo, Andre Poon, Molly Liepnieks, Kristy Harmon, Peter Moore

**Affiliations:** 1 Department of Pathology, Microbiology and Immunology, School of Veterinary Medicine, University of California, Davis, CA 95616-5270, USA; castillodiego@yahoo.com (D.C.); acpoon@ucdavis.edu (A.P.); mliepnieks@ucdavis.edu (M.L.); klmartinez@ucdavis.edu (K.H.); pfmoore@ucdavis.edu (P.M.); 2 Department of Veterinary Microbiology and Pathology, College of Veterinary Medicine, Washington State University, Pullman, WA 99163, USA; chrissy.eckstrand@wsu.edu

**Keywords:** FIV (feline immunodeficiency virus), lymphoma, FAIDS (feline AIDS), cat, immunopathology

## Abstract

Our laboratory has serially reported on the virologic and immunopathologic features of a cohort of experimental feline immunodeficiency virus (FIV)-infected cats for more than eight years. At 8.09 years post infection (PI), one of these animals entered the terminal stage of infection, characterized by undulating hyperthermia, progressive anorexia, weight loss, and pancytopenia; the animal was not responsive to therapeutic interventions, necessitating euthanasia six weeks later (8.20 years PI). Subsequent analyses indicated that neoplastic lymphocytes infiltrated multiple cervical lymph nodes and a band-like region of the mucosal lamina propria within a segment of the intestine. Immunohistochemistry and T cell clonality testing determined that the nodal and intestinal lesions were independently arising from CD3 T cell lymphomas. In-situ RNA hybridization studies indicated that diffuse neoplastic lymphocytes from the cervical lymph node contained abundant viral nucleic acid, while viral nucleic acid was not detectable in lymphocytes from the intestinal lymphoma lesion. The proviral long terminal repeat (LTR) was amplified and sequenced from multiple anatomic sites, and a common clone containing a single nucleotide polymorphism was determined to be defective in response to phorbol myristate acetate (PMA)-mediated promoter activation in a reporter gene assay. This assay revealed a previously unidentified PMA response element within the FIV U3 region 3’ to the TATA box. The possible implications of these results on FIV-lymphoma pathogenesis are discussed.

## 1. Introduction

Feline immunodeficiency virus (FIV) is a lentivirus that permissively infects CD4 T cells and a variety of other feline leukocytes, resulting in lifelong viral persistence and progressive immunopathology in infected cats. Long-term observation of both experimentally and naturally infected cats has shown that peripheral blood CD4 T cell counts progressively decrease over time, and that FIV-associated diseases eventually become apparent [1,2]. Potential consequences of FIV infection in cats include immune dysfunction, opportunistic infections, neoplasia, wasting, and death [3]. However, clinical outcomes in FIV-infected cats are variable, with many FIV-infected cats living approximately normal lifespans [4]. The complex factors contributing to these disparate outcomes of infection remain incompletely understood.

Perhaps due to the costs of keeping experimentally infected animals for protracted time periods, the acute and the early asymptomatic stages of infection have received the greatest investigative attention, while less is understood about FIV pathogenesis during the late asymptomatic period and the terminal stage of infection [5]. This terminal stage of infection is characterized by nonspecific clinical signs, followed by a stage akin to Acquired Immunodeficiency Disease Syndrome (AIDS), referred to as Feline AIDS (FAIDS) [6]. FAIDS is characterized by a variety of opportunistic infections, wasting, multiple neoplastic disorders, neurologic diseases, and cytopenias.

During FAIDS, FIV-infected cats have an increased incidence of neoplasia, with lymphoma (lymphosarcoma) representing the majority of these FIV-associated neoplasms [7]. FIV-infected cats are approximately five times more likely to develop lymphoma or leukemia than uninfected cats [8]. An increased incidence of lymphoma also occurs during the late stages of infection with primate immunodeficiency viruses HIV and SIV (simian immunodeficiency virus) in their respective hosts [9,10,11]. Although FIV-associated lymphomas have been typed as B cell, T cell or non-B/non-T cell origin [7], B cell lymphomas have most often been identified in both experimentally and naturally infected cats [12,13,14,15].

The mechanism(s) of FIV-associated lymphomagenesis remains controversial, with both direct and indirect mechanisms having been proposed [16]. This unresolved controversy is also true of the lymphoproliferative disorders associated with the primate lentiviral infections (HIV and SIV) in their respective hosts [16]. Although retroviruses from the Alpharetrovirus, Betaretrovirus, Gammaretrovirus, Deltaretrovirus, and Epsilonretrovirus genera are known to play a direct role in molecular oncogenesis, this is generally not thought to be the case for Lentiviruses. The majority of studies examining FIV-associated lymphoma have been most consistent with an indirect mechanism of tumorigenesis resulting from immunodeficiency-associated impaired immunosurveillance [7,13,17,18,19]. An argument has been made suggesting that co-infections with Gammaherpesvirus (*Felis catus* Gammaherpesvirus-1) may play a role in FIV-infected cat lymphomagenesis [16].

Here we describe the chronological and terminal virologic and immunopathologic features identified in a specific pathogen-free cat (cat 165) experimentally infected with FIV for more than eight years.

## 2. Materials and Methods

### 2.1. Animals and Pathology

Four specific pathogen-free (SPF) cats (animals 165, 184, 187, and 186) were intramuscularly inoculated with FIV-C-Pgmr viral inoculum at 5.5 months of age, as described previously [20]. Two age-matched SPF control animals (cats 183 and 185) were mock-inoculated with 1 mL of sterile culture media. After inoculation, the infected and control animals were continuously housed at the University of California (UC) Davis Feline Research Laboratory (FRL) as two separate cohorts, an FIV-infected group (*n* = 4) and uninfected group (*n* = 2). The animals were monitored for clinical illness by periodic physical examination, given daily behavior assessments (FRL staff), and sampled for blood by venipuncture approximately once per month. The study protocol was approved by the UC Davis Institutional Animal Care and Use Committee (IACUC #19930, permission date (10 May 2017)).

Humane criteria for euthanasia were established by the principal investigator (Murphy) in collaboration with Campus Veterinary Services, and were approved by IACUC. Under these criteria, an experimentally FIV-infected cat would be considered for euthanasia after prolonged anorexia or dehydration resulting in persistent and substantial loss of body weight (body condition score less than 4 out of 9), the occurrence of severe and persistent clinical signs (fever, neurologic signs, ophthalmologic signs, etc.), or the presence of severe hematologic abnormalities (persistent anemia resulting in a hematocrit at or below 15%, or hematologic malignancy, such as lymphoma or leukemia).

For cat 165, persistent pancytopenia, anorexia, and progressive weight loss justified euthanasia at 426 weeks (8.20 years) PI. Euthanasia was performed by intravenous administration of phenytoin sodium/pentobarbitol sodium (Beuthanasia, Merck, Kenilworth, NJ, USA) administered at >100 mg/kg body weight; a necropsy was immediately performed thereafter by one of the authors (M.L.). A complete set of tissues was collected and fixed in 10% buffered formalin. In addition, two additional sets of fresh tissue samples were collected, including brain, lymph node, bone marrow, small intestine, and spleen (<1 g tissue per sample). Tissues from one of these sets were packaged into individual Whirl-Pac bags (Nasco, Toronto, ON, USA) and archived at −70 °C for eventual DNA isolation. Frozen tissues were maintained at −70 °C for approximately one year prior to further processing (below).

Tissues from the other sample set were placed into individual sterile microcentrifuge tubes containing 1.5 mL RNAlater (Thermo Fisher Scientific, Waltham, MA, USA) and frozen at −70 °C for eventual RNA isolation. After nine days, formalin-fixed tissues were trimmed, placed in cassettes, routinely embedded, and processed for 5 μm-thick sections stained routinely with hematoxylin and eosin.

### 2.2. Isolation and Enumeration of Peripheral Leukocytes

The total number of peripheral white blood cells (total WBC) was serially determined as described previously, using either an automated Coulter Counter (Coulter ACT Diff, Beckman Coulter, Brea, CA, USA) or a LeukoCheck Kit (Biomedical Polymers, Inc., Gardner, MA, USA) and manual hemocytometer (Hausser Scientific, Horsham, PA, USA) [21,22]. The relative proportions of specific peripheral leukocyte subsets were determined by flow cytometry from 100 μL of whole blood, as described previously [22]. This procedure utilized the following antigen-specific antibodies, anti-feline CD4 (clone FE1.7B12), anti-feline CD8 (clone FE1.10E9), and anti-canine CD21 (B cells, clone CA2.1D6). These antibodies were obtained from the laboratory of Dr. P. Moore (UC Davis). Absolute cell counts were calculated by multiplying the total WBC count by the percent of cells expressing the specific antigen marker.

### 2.3. Isolation, Quantification, and Sequencing of Viral Nucleic Acids

Viral RNA was serially isolated and amplified by real-time PCR from clarified plasma, as described previously [22]. Quantification of plasma vRNA was based on a standard curve generated from viral transcripts, prepared by in-vitro transcription of a plasmid (pCR2.1, Invitrogen, Carlsbad, CA, USA) containing a 101 nucleotide FIV-C *gag* amplicon [20].

Nucleic acids were isolated from frozen feline tissues archived at the time of the necropsy examination (spleen, brain, small intestine, bone marrow, and lymph node). RNA was isolated from tissues that had been immersed in RNAlater (Thermo Fisher Scientific, Waltham, MA, USA) at the time of collection. For each tissue, approximately 30 mg of tissue was thawed on ice and mechanically disrupted, using a disposable Closed Tissue Grinder System (02-542-09, Fisher scientific, Waltham, MA, USA) in Buffer RLT with β-mercaptoethanol (Qiagen, Hilden, Germany). The disrupted tissue was subsequently homogenized using a QIAshredder column (Qiagen), and RNA was isolated with an RNeasy Kit (Qiagen), according to manufacturer’s instructions. Tissue-associated RNA was DNAse treated and reverse-transcribed to cDNA, as described previously [20].

DNA was isolated from approximately 25 mg of brain, small intestine, bone marrow, and lymph node, as well as from 10 mg of spleen. The tissues were thawed on ice in a sterile petri dish and placed into Buffer ATL (Qiagen), and minced using a sterile scalpel blade. Approximately 200 μL of this material was transferred to a sterile microcentrifuge tube with 20 μL proteinase K (Qiagen) and incubated at 56 °C for 3 h. Following digestion, homogenization was achieved by running the tissue mixture through a QIAshredder (Qiagen), and DNA was isolated using a DNeasy Blood and Tissue kit (Qiagen). The number of cells associated viral DNA and viral RNA (cDNA) was quantified and normalized to cellular GAPDH via real-time PCR, as described previously [20]. The real-time PCR assay has a detection limit of approximately 10 copies of FIV *gag*-complementary DNA (cDNA) per tissue sample, or 8 × 10^2^ copies of FIV *gag* cDNA per mL plasma [23].

The proviral long terminal repeat (LTR, promoter) was amplified and sequenced from multiple formalin-fixed paraffin-embedded (FFPE) tissues, including cervical LN1, cervical LN2, mesenteric LN, and bone marrow. The tissues were obtained from cat 165 during the necropsy examination and fixed in 10% formalin, sectioned into cassettes, and routinely processed into FFPE tissue blocks. Five μm thick sections of the FFPE tissues were cut and affixed to glass slides. For each tissue type, a sterile scalpel blade was utilized to harvest the specific tissue into a sterile microcentrifuge tube from five glass slides.

DNA was isolated from five serial sections of each tissue type (cervical LN1, cervical LN2, mesenteric LN, or bone marrow) using the QIAamp DNA FFPE Tissue Kit (Qiagen). DNA isolated from slides of FFPE cervical LN1 was assessed for the presence of *Felis catus* Gammaherpesvirus 1 by PCR, using methods described previously [24]. The proviral LTR was amplified through standard PCR using the primers FIV_U3for2_ (5′-GGA AGA TTA TTG GGA TCC TGA TG), FIV_U5rev_ (5′-TGC GAA GTC TTC GGC CCG GAC TCC G), and Taq polymerase (Invitrogen). The cycling conditions were 95 °C for 2 min, followed by 40 cycles of the following: 95 °C for 15 s, 55 °C for 30 s, and 72 °C for 30 s. A final amplicon extension was performed at 72 °C for 5 min. The resulting amplicons were directly sequenced from both the 5′ and 3′ directions by a local vendor, using the primers listed above. In order to assess sequence diversity, the amplicons derived from the cervical LN1 (lymphoma) were cloned using the TA Cloning Kit (Thermo Fisher Scientific, Waltham, MA, USA) and transformed into TOP10 competent *E. coli* bacteria (Thermo Fisher Scientific, Waltham, MA, USA). Twenty bacterial colonies were selected for the isolation of plasmid DNA clones (QIAprep Miniprep Kit, Qiagen) and submitted for sequencing by a local vendor.

### 2.4. Lymphocyte Antigen Receptor Gene Rearrangement Analysis

Clonality of lymphocyte antigen receptor genes (T cell receptor gamma locus (TRG)) was performed on tissues from cat 165, as previously described [25]. Briefly, DNA extraction was performed on 25 μm tissue sections that were cut from formalin-fixed paraffin-embedded (FFPE) tissue blocks using the DNeasy Extraction Kit (Qiagen), per the manufacturer’s instructions, slightly modified to include a 10-min incubation at 85 °C after the addition of AL buffer. Rearrangement of TRG was assessed by PCR amplification of the CDR3 region between the V and J genes. Briefly, 100 ng of genomic DNA from each sample (cervical lymph node and small intestine) was amplified using a consensus primer derived from TRG V genes (5′ -GAAGAGCGAYGAGGGMGTGT-3′, 20 pmol) in conjunction with a consensus primer derived from TRG J genes (5’ -CTGAGCAGTGTGCCAGSACC-3′, 10 pmol). Specific amplicon size was expected to occur within a target range of about 80–120 base pairs. Amplification conditions used a two-step, modified touchdown protocol to increase specificity of the reactions. All PCR reactions were run in triplicate.

PCR products were size-separated using an eGene HDA-GT12 capillary electrophoresis analyzer (renamed QIAxcel, Qiagen). The results were in the form of a histogram view (fluorescence intensity versus time/amplicon size) or a gel view (a computed pseudogel image). Clonal rearrangements produced a “peak” or “band”, and polyclonal rearrangements produced a “curve” or “smear” in the histogram and gel view, respectively. Positive and negative control clonality samples were run in parallel, and were comprised of a known feline lymphoma lesion derived from a FIV-negative cat (positive control) and normal lymphoid tissue from a different FIV-negative cat (negative control).

### 2.5. In-Situ Hybridization and Immunohistochemistry Studies

An in-situ hybridization technique was used to visualize the presence, location, and quantity of tissue associated viral nucleic acid in the following tissues: cervical LNs 1 and 2 (lymphoma), mesenteric LN (lymphoid atrophy), small intestine, and bone marrow. The assay was performed on 5 μm sections of formalin-fixed paraffin-embedded tissue set on positively charged glass slides, according to manufacturer protocols, using the RNAscope 2.5 Brown kit (Advanced Cell Diagnostics, Newark, CA, USA) and a nucleic acid probe designed to hybridize FIV-C-specific RNA (cat# 462951) [5]. Spleen, lymph node, and gut tissues from an age-matched sham-infected SPF cat were used as a negative control [5]. A probe specific for a feline housekeeping gene RNA (*Felis catus* peptidylprolyl isomerase B) and for the *Bacillus subtilis* strain SMY dihydrodipicolinate reductase (dapB, cat #310043) gene were used as positive and negative controls, respectively.

Immunohistochemistry (IHC) assays detecting CD3 antigen (clone CD3-12, P. Moore, Davis, CA, USA) were performed on 5 μm-thick, FFPE tissue sections (cervical LN 1 and 2, mesenteric lymph node, small intestine, and bone marrow) using a streptavidin biotin detection system (Biocare Medical, Pacheco, CA, USA), as described previously [26].

### 2.6. Reporter Gene Assays

In order to assess the putative effect of the U3 _G_196_A_ single nucleotide polymorphism (SNP) on FIV promoter functionality, a reporter gene assay was performed. This β-galactosidase reporter gene system has been utilized previously in our laboratory to assess viral promoter functionality [21]. In this system, the inoculating sequence of the FIV LTR was situated 5′ to the reporter gene β-galactosidase within the plasmid construct pLTR Blue. The plasmid pLTR Blue was subsequently modified (mutagenized) to include the U3 _G_196_A_ SNP, using a site-directed mutagenesis kit, according to the manufacturer’s instructions (QuikChange Lightning Site-Directed Mutagenesis Kit, Agilent Technologies, ‎Santa Clara, CA, USA). The resulting plasmid (pLTR _G_196_A_ Blue) was sequenced to confirm the substitution of adenine for guanine at position 196 of the FIV promoter.

Reporter gene assays were performed as described previously [27], with the following exceptions: 293T cells were transfected in a six-well plate format, using 2 μg of plasmid DNA and 4 μL of jetPRIME^®^ transfection reagent (Polyplus-transfection^®^ SA, New York, NY, USA), following the manufacturer’s protocol. Cells were transfected with the original reporter plasmid pLTR Blue, or pLTR _G_196_A_ Blue, with or without exposure to the mitogen phorbol myristate acetate (PMA, Sigma-Aldrich, St. Louis, MO‎, USA). PMA was added 4 h post-transfection at a concentration of 10 μM. Control plasmids included pCMV Blue (constitutive promoter, positive control), and pBlue empty (no promoter, negative control). Cells were harvested 24 h post-transfection, and the β-galactosidase assay was performed following a published protocol (“Protocol 7: Assay for β-galactosidase in Extracts of Mammalian Cells”) [28]. Each experiment was performed three times in concurrent triplicate culture wells.

### 2.7. Inverse PCR

Inverse PCR was performed according to the protocol established by Sambrook and Russell [29]. Briefly, DNA was extracted from five serial sections of formalin-fixed paraffin-embedded lymphoma tissue using the QIAamp DNA FFPE Tissue Kit (Qiagen). One μg of DNA was digested with BamHI restriction endonuclease (New England BioLabs, Ipswich, MA, USA). The BamHI-digested DNA was self-ligated into rings using the Quick Ligase Kit (NEB). Ring-ligated DNA was subsequently purified using Amicon Ultra, using 0.5 mL 100 k centrifugal filter units (Millipore, Burlington, MA, USA). The proviral integration site was amplified through standard PCR using Q5 High-Fidelity DNA Polymerase (NEB), and the primers FIV U5 for (5′-GGGCCGAAGACTTCGCA) and FIV RNA 3′ (5′-TGGAACAAATCTACGTCATCGG). In inverse PCR, the primers are directed *away* from one another (see [29], page 8.81). The PCR cycling conditions were as follows: 98 °C for 30 s, followed by 35 cycles of 98 °C for 10 s, 52 °C for 30 s, 72 °C for 3 min, and a final elongation step of 72 °C for 2 min. The resulting PCR product was ligated into pCR2.1 using Invitrogen’s TA Cloning Kit, following manufacturer’s protocol, and transformed into One Shot TOP10 Chemically Competent *E. coli.* Clones were cultured overnight at 37 °C on LB plates containing X-gal for blue/white screening. Thirty white clones were selected and propagated in 5 mL of LB broth containing Carbenicillin, and placed in a 37 °C shaker incubator for 12 h. Plasmid minipreps were isolated from each clone using a Zyppy Plasmid Miniprep Kit (Zymo Research, Irvine, CA, USA) and sequenced through a local vendor (DNA Sequencing Facility, UC Davis, Davis, CA, USA).

### 2.8. Statistical Tests

The graphical numerical data is presented as the mean of three or more values, with the standard deviation or range represented by error bars. Statistical differences were determined by unpaired Student’s *t*-tests. A *p* value of <0.05 was considered to be statistically significant. Statistics were performed with Prism 6 software (GraphPad Software, Inc., La Jolla, CA, USA).

## 3. Results

### 3.1. Clinical Trajectory

**Figure 1:**
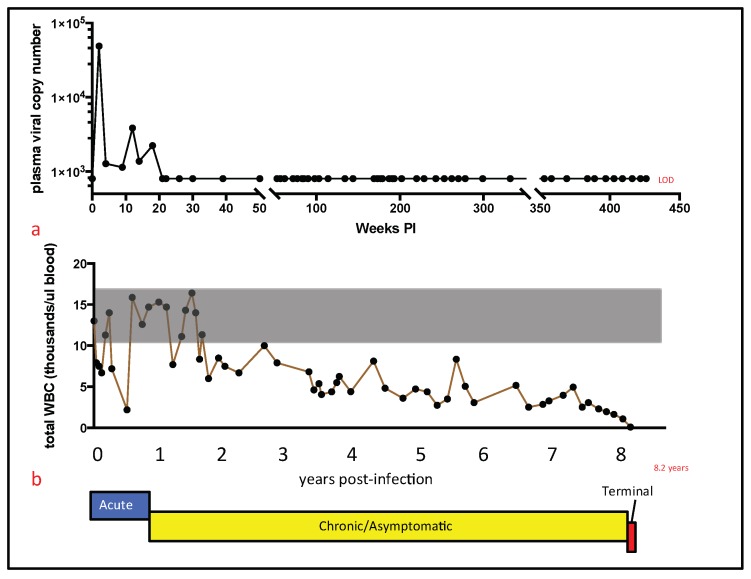
Plasma viral copy number, total white blood cells (WBC), and clinical trajectory of cat 165. (**a**) The plasma viral copy number is plotted as number of copies *gag* RNA/mL blood vs. weeks post-infection (PI). The limit of detection (LOD) is approximately 800 copies vRNA/mL blood; (**b**) The total number of WBC (in thousands of leukocytes/microliter blood) is plotted against years PI—8.2 years (red X) is the time of euthanasia PI. The clinical trajectory of FIV for cat 165 is depicted below the x-axis- acute stage (blue), asymptomatic stage (yellow) and terminal stage (red).

The clinical trajectory of FIV-associated disease for cat 165 is depicted graphically in Figure 1b. The acute stage lasted approximately 10 months PI, and was delineated by the initial presence of a detectable plasma viremia (2–18 weeks PI); transient, bilateral lymphadenomegaly of the popliteal lymph nodes (3–9 months PI); and recurring, multifocal cutaneous bacterial infections (culture positive for *Streptococcus canis*) on the dorsum, chin, ears and digits (3–10 months PI). Despite these maladies, cat 165 maintained a strong appetite, manifested normal social behaviors, and maintained a healthy body weight throughout the acute stage.

The subsequent asymptomatic stage was typified by a generalized lack of overt clinical morbidity, and lasted from ~10 months PI until the onset of the terminal stage of the disease, ~8.09 years PI. Despite the lack of overt morbidity during this stage, multiple peripheral leukocytic subsets progressively and markedly declined throughout the asymptomatic stage. The terminal stage of the infection (non-specific/FAIDS) was relatively brief, lasting approximately six weeks, and was characterized by profound panleukopenia, nonregenerative anemia (hematocrit of 20–26), undulating fever, progressive weight loss (11.5% body weight), and progressive anorexia. A variety of diagnostic tests (chemistry panels, urinalyses, thoracic radiography, and abdominal ultrasound) were not consistent with a specific disease process. The progressive nature of the weight loss, anorexia, anemia, and panleukopenia, as well as a general lack of a response to symptomatic therapy, fulfilled the criteria for euthanasia.

### 3.2. Plasma Viral Copy Number and Peripheral Leukocyte Numbers

For cat 165, plasma viral RNA (vRNA) was initially detectable during the acute stage of infection, but became undetectable thereafter. Plasma vRNA quantification by a real-time reverse transcriptase (RT)-PCR assay for cat 165 revealed peak viremia at two weeks post-infection, with 4.9 × 10^4^ copies of *gag* cDNA/mL plasma, and was detectable until 18 weeks PI (Figure 1a). Plasma vRNA was not detected at any time point for the remainder of the cat’s life (a total of 45 real-time PCR assays distributed over 7.8 years). The final assessment of plasma viremia was made at 422 weeks (8.09 years) PI. Notably, plasma viremia was not detected at this time point. Euthanasia was performed approximately one month later, at 426 weeks PI (8.20 years).

**Figure 2:**
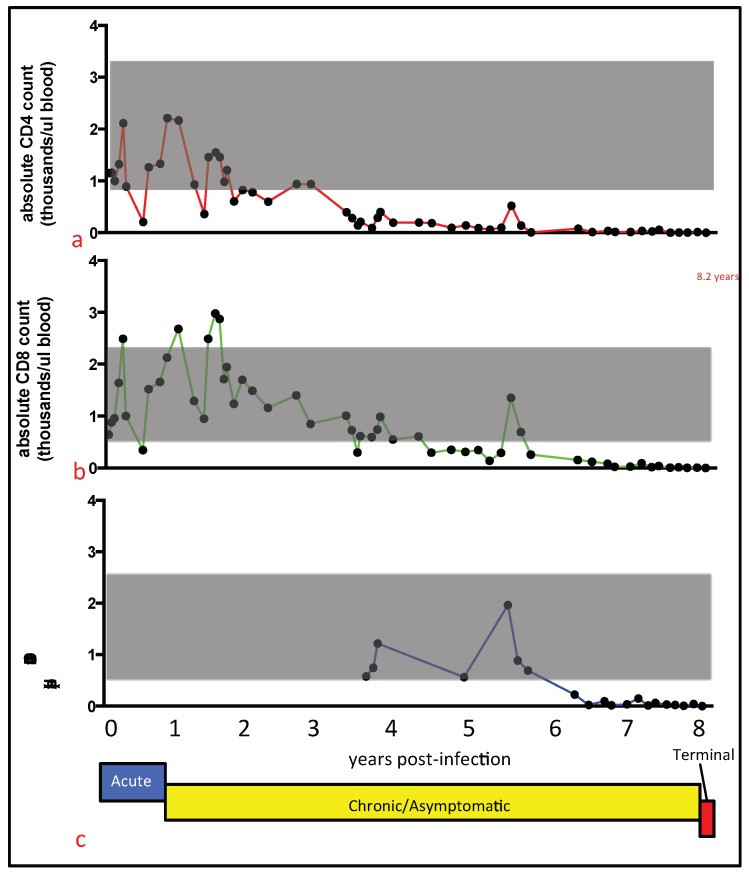
The absolute number of peripheral CD4, CD8, and CD21 cells progressively declined over the course of the infection. (**a**) CD4, (**b**) CD8, and (**c**) CD21 cells are graphed in thousands of cells/microliter blood vs. time PI (years). The grey shaded regions represent the mean numbers of leukocytes ± standard deviation for the two uninfected control cats during the eight-year study period. The clinical trajectory of FIV for cat 165 is depicted below the x-axis for Figure 2c, acute stage (blue), asymptomatic stage (yellow) and terminal stage (red).

The absolute numbers of peripheral white blood cells, as well as CD4, CD8, and CD21 cells progressively declined over 8.20 years of FIV infection (Figure 1 and Figure 2). This finding is perhaps remarkable in light of the apparent clinical health of cat 165 during much of this time (asymptomatic stage). The total number of white blood cells (T WBC) is plotted in Figure 1b over the entire time cat 165 was infected with FIV. From approximately two years PI onward, the T WBC number was less than the mean ± standard deviation of the T WBC count for the two uninfected control animals (grey shaded region). Approximately three weeks prior to euthanasia, the T WBC count was only 90 cells/microliter. Peripheral CD4, CD8, and CD21 cells progressively and markedly declined during the asymptomatic stage of infection (Figure 2a–c, respectively). Peripheral CD4, CD8, and CD21 cells fell below 500 cells/microliter at ~4, 5.5, and 6.5 years PI, respectively, and did not recover to normal levels.

### 3.3. Pathological Findings

Minimal gross changes were identified during the gross necropsy examination. Subcutaneous adipose tissue and muscling was adequate. Submandibular and cervical lymph nodes (LNs) were identified and considered to be grossly normal, while other peripheral, abdominal, and thoracic LNs were not definitively identified. Numerous small pale nodules were diffusely distributed throughout the pancreatic parenchyma (nodular hyperplasia). The pole of the left renal cortex was focally collapsed (chronic renal infarct), and the left renal crest had an irregular margin.

Microscopically, alimentary (stomach, intestine, cecum, and colon), and spleen-associated lymphoid tissues were diffusely atrophied. Pancreatic islets had diffuse and severe amyloidosis. Bilateral thyroid glands had adenomatous nodular hyperplasia. Bilateral kidneys had moderate and diffuse lymphocytic/plasmacytic interstitial nephritis and chronic focal infarction.

**Figure 3:**
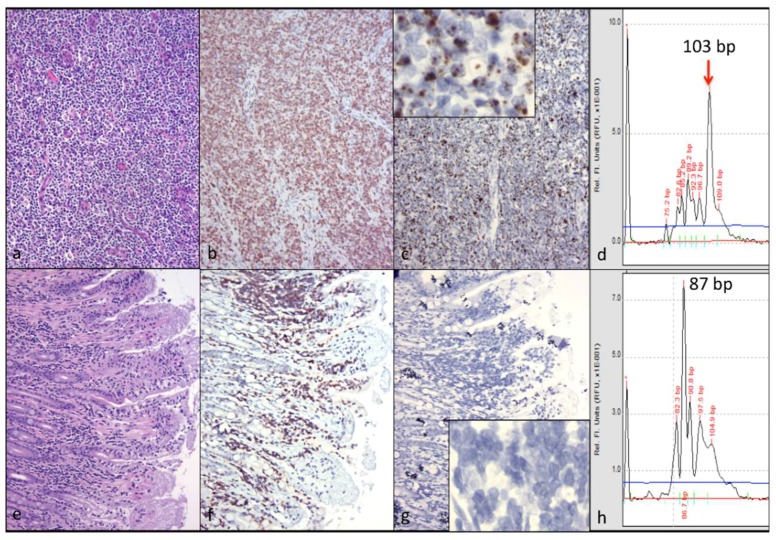
Clonally distinct, CD3+ T cell lymphomas are present in the cervical lymph nodes and the mucosa of the small intestine (jejunum). (**a**) The architecture of the cervical lymph node (LN) is effaced by sheets of neoplastic lymphocytes (hematoxylin and eosin stain); (**b**) The neoplastic lymphocytes within the cervical LN are CD3+ T cells (CD3 IHC); (**c**) Diffuse neoplastic lymphocytes within the cervical LN contain brown aggregating granules (positive for viral nucleic acid, in-situ hybridization); inset shows high-power magnification of neoplastic lymphocytes demonstrating brown granular material (viral nucleic acid); (**d**) A monoclonal PCR amplicon derived from the cervical lymphoma lesion is 103 base pairs (bp) long (T cell receptor gamma locus (TRG) clonality); (**e**) A poorly-delineated band of lymphocytes infiltrates the base of the mucosal villi within a segment of jejunum (hematoxylin and eosin stain); (**f**) The infiltrating lymphocytes are CD3+ (CD3 IHC); (**g**) Essentially, no brown granular material is present in these infiltrating lymphocytes (undetectable viral nucleic acid). Inset: high-power magnification; (**h**) A monoclonal PCR amplicon derived from the intestinal lesion is 87 bp long (TRG clonality).

Although the cervical lymph nodes were not grossly enlarged, histologically, the architecture of the node (cervical LN1) was replaced by neoplastic lymphocytes forming homogeneous sheets effacing the node (Figure 3a). A CD3 IHC stain revealed these neoplastic lymphocytes to be CD3+ T cells (Figure 3b). A CD20 IHC stain revealed small numbers of remnant B cells scattered throughout the neoplastic T cells. In an FIV in-situ hybridization assay, the majority of the neoplastic T cells contained variably-sized aggregates of brown granular material, consistent with intracellular viral nucleic acid (Figure 3c). Photomicrographs of positive and negative control slides for the in-situ hybridization assay are available as supplementary figures (Figures S1 and S2).

TRG clonality analysis of DNA obtained from sections of this cervical lymph node demonstrated a monoclonal amplicon of 103 bp, consistent with a monoclonal VDJ (variable- D-joining) rearrangement and CD3 lymphoma (Figure 3d). These findings were similar in the other examined cervical lymph node (cervical LN2), and in the LN tissue utilized for DNA extraction indicating the same clonal neoplastic process was present in at least three cervical lymph nodes.

For the alimentary tissues, two sections of stomach, five sections of small intestine, one section of cecum, and two sections of colon were examined histologically. In a discreet segment of small intestine (two sections of jejunum), a circumferential band within the lamina propria and at the base of the villi was infiltrated by lymphocytes (Figure 3e) that were shown by IHC to be CD3 cells (Figure 3f). Interestingly, an FIV in-situ hybridization assay demonstrated essentially no detectable viral nucleic acid within these lymphocytes (Figure 3g). On the same slide as the intestinal lesion, scattered lymphocytes within the submucosal lymphoid tissue (Peyer’s patches) contained brown granules (viral nucleic acid, in slide positive control). TRG clonality analysis of DNA samples isolated from this segment of small intestine demonstrated a monoclonal amplicon of 87 bp (Figure 3h). Collectively, these results are consistent with a unique clone of CD3+, which is viral nucleic acid-independent, mucosal lymphoma. Photomicrographs of a histologically normal section of small intestine are provided for comparison as supplementary figures (Figures S3 and S4).

**Figure 4:**
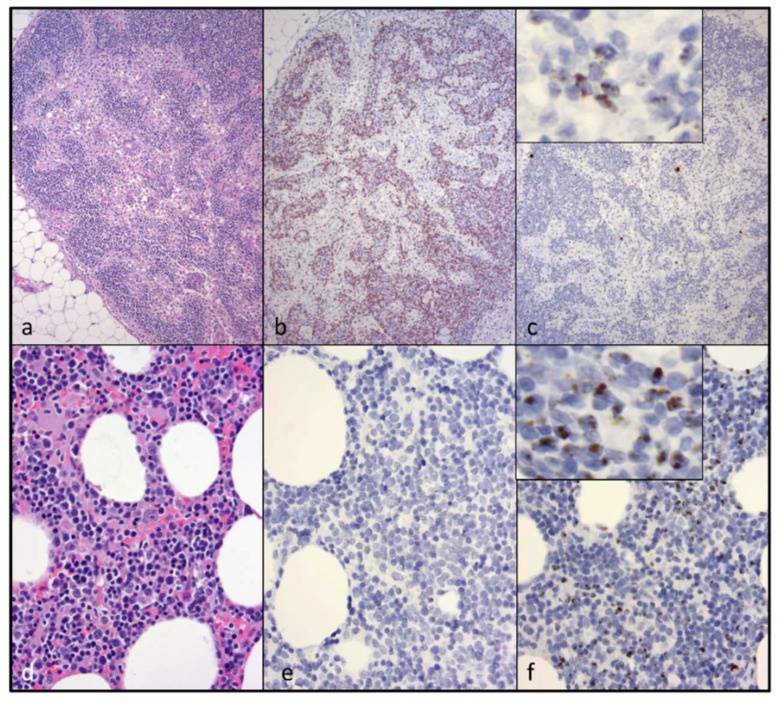
An atrophied mesenteric lymph node (LN) and bone marrow contain moderate amounts of viral nucleic acid, but are variably positive for CD3+ T cells. (**a**) The mesenteric LN has a poorly developed cortex lacking follicles (cortical/follicular atrophy, H&E); (**b**) The remnant cortex and medullary cords have moderate numbers of CD3+ T cells (non-neoplastic lymphocytes, CD3 IHC); (**c**) Scattered leukocytes in the mesenteric LN contain moderate amounts of brown granular material—inset shows high-power magnification (viral nucleic acid, in-situ hybridization); (**d**) Bone marrow (BM) is histologically normal (hematoxylin and eosin stain); (**e**) CD3+ T cells are not detectable in the BM (CD3 IHC); (**f**) Scattered hematopoietic cells in the BM contain brown granular material (viral nucleic acid, in-situ hybridization).

A mesenteric lymph node demonstrated atrophied cortical follicles and medullary cords (Figure 4a). Scattered to coalescing lymphocytes within the cortex and medullary cords were revealed by IHC to be CD3+ T cells (Figure 4b) and CD20+ cells. An in-situ hybridization assay indicated that scattered and aggregating lymphocytes within the mesenteric lymph node contained small amounts of intracellular viral nucleic acid (Figure 4c). A sample of bone marrow was considered by IHC to be histologically normal (Figure 4d) and diffusely CD3 negative (Figure 4e). An in-situ hybridization assay demonstrated moderate numbers of hematopoietic cells containing viral nucleic acid (Figure 4f).

### 3.4. Tissue Proviral and Viral RNA Loads

**Figure 5:**
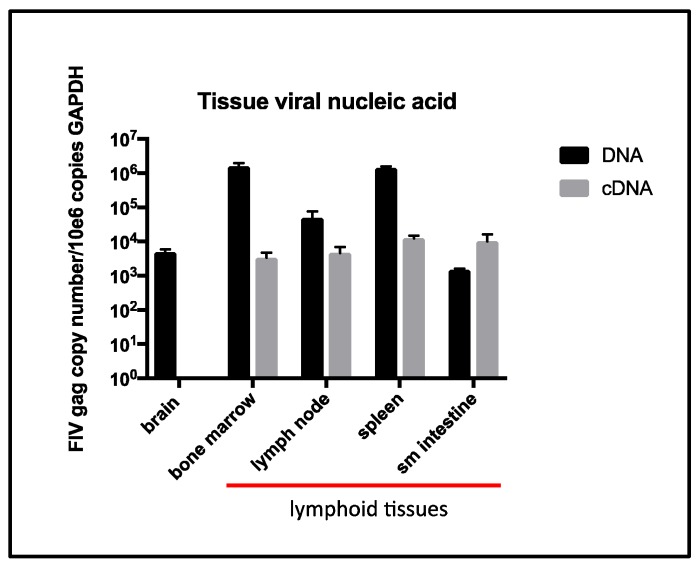
Proviral DNA (black bars) is detectable by real-time PCR in all of the examined feline tissues while vRNA (cDNA, grey bars) is only detectable in lymphoid tissues (red line).

The quantity of proviral DNA isolated from various tissues (brain, bone marrow, lymph node, spleen, and small intestine) varied between ~1 × 10^3^ and 1 × 10^6^ copies FIV *gag* per 1 × 10^6^ copies GAPDH (black bars, Figure 5). The quantity of vRNA (cDNA) isolated from various tissues (bone marrow, lymph node, spleen, and small intestine) varied between ~1 × 10^3^ and 1 × 10^4^ copies FIV *gag* per 1 × 10^6^ copies GAPDH (grey bars, Figure 5). Tissue-associated vRNA (cDNA) was not detectable in brain tissue, although abundant tissue-associated GAPDH RNA was detected. *Felis catus* Gammaherpesvirus was not identified through a PCR assay of DNA extracted from cervical LN1.

### 3.5. Promoter Sequence Diversity and Reporter Gene Assay

**Figure 6:**
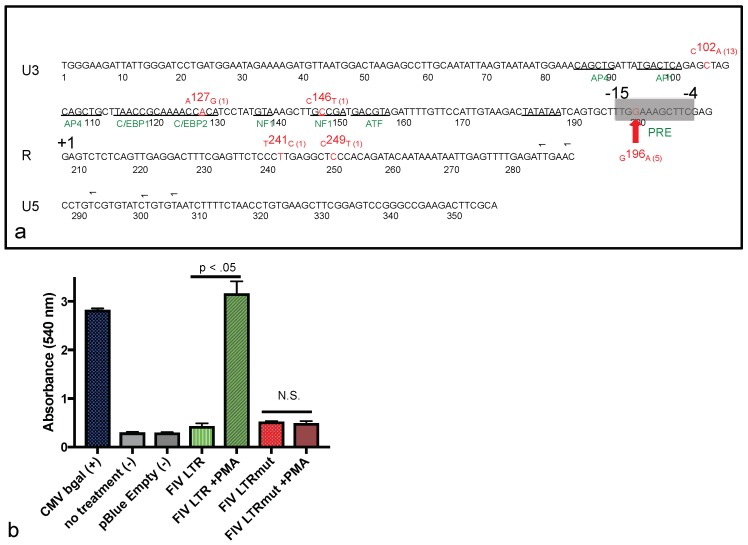
(**a**) The FIV promoter (LTR) sequenced from the tissues of cat 165 has multiple single nucleotide point (SNP) mutations. The U3, R, and U5 regions of the inoculating FIV sequence are depicted in black, while the various SNPs are indicated in red; parentheses indicate the number of times the SNP was isolated out of 14 sequenced clones. Known TFB sites are indicated in green, and the putative phorbol myristate acetate (PMA) Response Element (PRE) is denoted by a shaded grey box (nucleotide position −15 to −4, where +1 is the viral transcription start site, R). The _G_196_A_ SNP is emphasized with a red arrow; (**b**) A β-galactosidase reporter gene assay identifies a novel PMA Response Element in the 3′ aspect of the FIV U3 region. The basal function of the wild type promoter (FIV LTR, green) and FIV promoter with the _G_196_A_ SNP (FIV LTRmut, red) is minimal relative to negative controls (no treatment and pBlue Empty). PMA treatment markedly induces the wild-type FIV promoter expression (FIV LTR + PMA, *p* < 0.05, green cross-hatch), but has no detectable effect on the FIV promoter with the _G_196_A_ SNP (FIV LTRmut, not significant, red cross-hatch), relative to transfected cells not treated with PMA.

The proviral LTR sequences derived from cat 165 tissues (cervical LN1/LN2, mesenteric LN and bone marrow) are depicted in Figure 6a. The provirus derived from all four of these tissues (direct sequenced PCR amplicons) concurrently demonstrated both the _C_102_A_ and _G_196_A_ SNPs.

In order to more precisely determine the frequency of the viral promoter sequence diversity derived from one of these tissues (cervical LN1/lymphoma), we cloned the PCR amplicons in *E. coli*, amplified the plasmid DNA, and attempted to sequence 20 individual plasmid clones. Fourteen of these twenty clones yielded interpretable sequences. In decreasing order of frequency, the _C_102_A_ SNP was identified in 13 of 14 clones, _G_196_A_ (five clones), _A_127_G_ (one clone), _C_146_T_ (one clone), _T_241_C_ (one clone), and _C_249_T_ (one clone) (Figure 6a).

The _C_102_A_ SNP has been previously isolated from the tissues of cat 165. Our group has demonstrated that this SNP does not alter FIV promoter function in a reporter gene assay [20]. The _G_196_A_ was a novel point mutation that had not been previously isolated from cat 165 or any of the other FIV-infected animals in this cohort. In order to test the hypothesis that the _G_196_A_ SNP results in a gain of function (increased viral promoter activity), we introduced this viral promoter sequence into a β-galactosidase reporter gene assay.

In this assay, the basal promoter function of the _G_196_A_-containing promoter (FIV LTRmut) was weak, similar to the wild-type FIV promoter (FIV LTR) (Figure 6b). However, when transfected cells were stimulated with the mitogen PMA, the wild-type FIV promoter was markedly activated (green bar), while PMA-associated induction was not evident with the mutated promoter (red bar). In this assay, the constitutively active CMV promoter (CMV bgal) served as a positive control, while no transfection (no treatment) and a β-galactosidase construct lacking a promoter (pBlue Empty) served as negative controls. This assay was repeated in triplicate wells three times, with similar results. These promoter function results suggest the presence of a previously unidentified transcription factor-binding motif within the 3′ aspect of the FIV U3 region. A search using the JASPAR database (jaspar.genereg.net) revealed the putative presence of a PMA Response Element, 5′-TTGGAAAG^A^/_C_^C^/_T_TTC-3′ (Figure 6a, PRE, grey region, nucleotides −15 to −4). The _G_196_A_ results in a G to A polymorphism at the second G in this sequence motif. The functional result of the other less commonly-identified SNPs (_A_127_G_, _C_146_T_, _T_241_C_, and _C_249_T_) on viral promoter function was not examined.

### 3.6. Inverse PCR

Thirty sequenced plasmid clones yielded no FIV sequences. Cloned sequences included fragments of bacterial and *Felis catus* genomes.

## 4. Discussion

Recent and detailed studies of the terminal stage of FIV infection have been reported infrequently in the FIV literature. Here we report on the chronological and terminal virologic/pathologic findings in an experimentally FIV-infected cat euthanized during the terminal stage of infection. In this FIV-infected cat, progressive leukocytopenia, anemia, undulating fever, anorexia, and weight loss necessitated euthanasia. Subsequent gross necropsy and microscopic examinations revealed a number of relatively common lesions of aging (>8 year old) cats, including interstitial nephritis, focal renal infarction, islet amyloidosis, and pancreatic/thyroid gland nodular hyperplasia. Diffuse islet amyloidosis has been associated with but is not necessarily a cause of feline diabetes mellitus. We considered all of these feline lesions to be age-related, and to be incidental/subclinical as clinical evidence of renal, thyroid, or pancreatic disease was not identified from the antemortem serum chemistry and urinalysis assays.

CD3+ T cell lymphoma was identified in both of the examined cervical lymph nodes and in a discrete segment of the intestinal mucosa, but was not identified in the atrophied mesenteric lymph nodes or other examined lymphoid tissues (spleen and bone marrow). At the time of the gross necropsy examination, lymphoma was not suspected, as lymphadomegaly was not identified. Although feline nodal lymphoma can result in node enlargement, this is not always the case (especially early on in the disease course). Obliteration of the nodal architecture by sheets of a monomorphic population of CD3 T cells, in conjunction with an amplified monoclonal T cell receptor, are consistent with a diagnosis of lymphoma. Segmental infiltration of the intestinal mucosa by small, differentiated neoplastic lymphocytes that fail to disrupt or distort the intestinal wall is a relatively common feline lesion [25]. The diagnosis of small T cell intestinal lymphoma is further supported by IHC and TRG clonality results. Notably, three of the authors are veterinary anatomic pathologists (B.G.M., P.M., and C.E.), while one of the authors is an anatomic pathology resident (M.L.).

The contribution of the nodal and intestinal lymphoma lesions to the clinical signs of pancytopenia, non-regenerative anemia, anorexia, progressive weight loss, and fluctuating fever was not determined. Notably, evidence of lymphoma was not identified histologically in the examined sections of bone marrow. However, fever, anorexia, and weight loss are known to be associated with feline lymphoma.

While abundant viral nucleic acid was evident by in-situ hybridization within the neoplastic lymphocytes of the cervical lymphoma lesion, viral nucleic acid was not detectable in the intestinal lymphoma lesion. We had previously determined that this FIV in-situ hybridization assay is highly sensitive; in our hands, it is perhaps more sensitive than tissue-based real-time PCR [5]. We wished to determine if the cervical and intestinal lymphoma lesions were pathogenically related. Lymphocyte PCR clonality assays demonstrated that the LN and intestinal lymphomas were clonally distinct. Although the tissues from cat 165 were fixed in formalin for a protracted period of time (nine days), the moderate to abundant amounts of viral RNA detectable in several different tissues (cervical lymph nodes, bone marrow, and mesenteric lymph nodes) suggested that the in-situ hybridization assay was not markedly affected. Viral nucleic acid was also detectable in the submucosa of lymphoma-affected sections of the small intestine (Peyer’s patches).

We considered (hypothesized) the possibility that the FIV lentivirus might be playing a direct role in a neoplastic pathogenesis, mediated through the viral integration site within the feline genome or through an activating mutation within the viral promoter. The basis for this hypothesis was the stark disparity in the in-situ hybridization findings. We wondered if the neoplastic lymphocytes in the cervical lymphoma lesion were filled with abundant viral nucleic acid, because the lymphocytes happen to be appropriately infectible by FIV (“fertile soil”)—or alternatively, did the lymphocytes represent a clonal expansion of a transformed T cell population that was infected early on or prior to transformation? The latter possibility would provide support for a direct role for viral oncogenesis. Utilizing the limited quality and amount of DNA obtainable from the FFPE tissues, we attempted to perform inverse PCR in order to determine the lesion-associated viral integration site(s) within the feline genome and whether or not this site was clonal. Notably, clonal integration of the FIV genome has been previously demonstrated in only a single case [30]. Unfortunately, these inverse PCR experiments were not successful.

We successfully amplified and sequenced the viral promoter from multiple individual FFPE tissues. Perhaps due to the undetectable presence of viral nucleic acid in the intestinal lymphoma lesion (via in-situ hybridization), we were not able to amplify and sequence the viral promoter from this tissue. We identified two relatively common SNPs within the viral promoter (LTR U3 region), isolated from a variety of tissues from cat 165. One of these SNPs, _G_196_A_, was a novel point mutation that had not been previously isolated from cat 165 or any of the other FIV-infected animals in his cohort. In a reporter gene assay, we determined that the _G_196_A_ SNP resulted in (i) minimal basal promoter function, similar to the wild-type FIV promoter (inoculating sequence); and (ii) a complete loss of promoter response to PMA-associated induction. The phorbol ester PMA has long been used as a potent activator of the related small ruminant lentiviral promoter [31,32,33,34]. Contrary to our hypothesis, we failed to identify a common viral mutation associated with augmented promoter function. However, a putative PMA Response Element (PRE) was identified surrounding the _G_196_A_ SNP through the use of the online JASPAR database (5′-TTGGAAAG^A^/_C_^C^/_T_TTC-3′, at nucleotides −15 to −4). The signaling mechanism and identity of the putative transcription factor(s) mobilized through PMA-induced activation of the FIV PRE were not determined here. Sparger, et al. have previously demonstrated that the FIV promoter in some viral strains is activated by a combination of PMA (50 ng/mL) and phytohemagglutinin (2 microgram PHA/mL) up to 10 fold above basal levels [35].

The potential identification of a novel transcription factor binding site is noteworthy. Approximately 30 years after the discovery of FIV, the transcriptional control of this virus, and how transcriptional regulation relates to viral production and latency, remains incompletely understood. Viral reactivation from latency strategies (antilatency therapies, or ALT), modeled in FIV-infected cells and cats, have been attempted [36,37]. Going forward, a more sophisticated understanding of the transcriptional regulation of this lentivirus will facilitate successful and practical ALT in FIV-infected cats.

The terminal tissue proviral loads for cat 165 were elevated from assays performed previously (antemortem surgical biopsies obtained ~6 years PI from cat 165) for spleen, small intestine [5], and peripheral popliteal lymph nodes biopsied ~5 years PI [26]. However, terminal tissue viral RNA loads were similar for the intestine and spleen relative to the same tissues sampled at ~6 years PI [5], but were less than the peripheral lymph nodes sampled ~5 years PI [26]. Interestingly, a terminal brain tissue sample contained abundant proviral DNA, while viral RNA (cDNA) remained undetectable. The tissues in which viral RNA was detectable (LN, spleen, intestine, and bone marrow) all contain resident populations of lymphoid cells, consistent with the concept that FIV replication occurs most readily in leukocytes.

Although a terminal blood sample was not obtained from cat 165, a blood sample obtained four weeks prior to euthanasia had undetectable plasma viremia. Although examination of a terminal blood sample would have been optimal, our collective data described here provide essentially no evidence of increased tissue or peripheral viral replication during the terminal stage of FIV infection. These findings run counter to dogma that viral loads increase terminally as the CD4 T cell number progressively falls in FIV-infected cats. Considering the profound and persistent leukopenia, it is also somewhat remarkable that we found no gross or histologic evidence for infectious or bone marrow disease. This may be related to the protected environment in which cat 165 was housed and limited sampling of the marrow.

In aggregate, our results support the conclusion that FIV likely played an indirect role in lymphomagenesis in cat 165. This assessment is based upon several lines of evidence. No viral promoter-activating mutation was amplified from the lymphoma tissue. The stark disparity in the amount of viral nucleic acid identified through in-situ hybridization associated with the nodal lymphoma versus the intestinal lymphoma suggests that lymphoma can occur in the absence of detectable viral nucleic acid. Finally, no detectable increase in viral RNA was identified in lymph node any other tissues, relative to prior samples from cat 165.

## 5. Conclusions

Conclusions from this study include the following:

- Despite a progressive and profound loss of peripheral CD4, CD8, and CD21 cells, we found no evidence of tissue or peripheral viral replication during the terminal stage of infection.
- We found no evidence of infectious disease(s) during the terminal stage of infection.
- Clonality assays identified independently arising T cell lymphomas in the cervical lymph nodes and gut.
- In-situ hybridization studies provide novel information on viral nucleic acid cell and tissue distribution. Although intriguing, the large disparity in detectable FIV nucleic acid in the cervical lymphoma versus the intestinal lymphoma likely reflects viral opportunism in taking advantage of available host lymphocytes, and not a direct cause of lymphoma (i.e., through insertional mutagenesis).
- These data suggest that FIV likely played an indirect role in the pathogenesis, leading to the independent emergence of lymphoma in the gut and cervical lymph nodes. This interpretation of an indirect mechanism of FIV-associated tumorigenesis (i.e., resulting from impaired immunosurveillance) is compatible with the majority of prior studies focused on FIV pathogenesis.
- Discovery of a novel PMA-activated motif within the 3′ U3 region warrants further investigation.

## Supplementary Materials

The following are available online at http://www.mdpi.com/1999-4915/10/6/280/s1, Figure S1: Photomicrograph of in-situ hybridization positive control slide (PPID gene, adrenal gland, cat 165, 600× magnification); Figure S2: Photomicrograph of in-situ hybridization negative control slide (bacterial DapB, adrenal gland, cat 165, 600× magnification); Figure S3: Histologically normal section of small intestine (cat 165, 20× magnification); Figure S4: Histologically normal section of small intestine (cat 165, 100× magnification).
