# Supplementary figures and images for: Multiple, Independent T Cell Lymphomas Arising in an Experimentally FIV-Infected Cat during the Terminal Stage of Infection
Source: Viruses. 2018 May 24;10(6):280. doi: 10.3390/v10060280 (PMC6024646; doi:10.3390/v10060280)

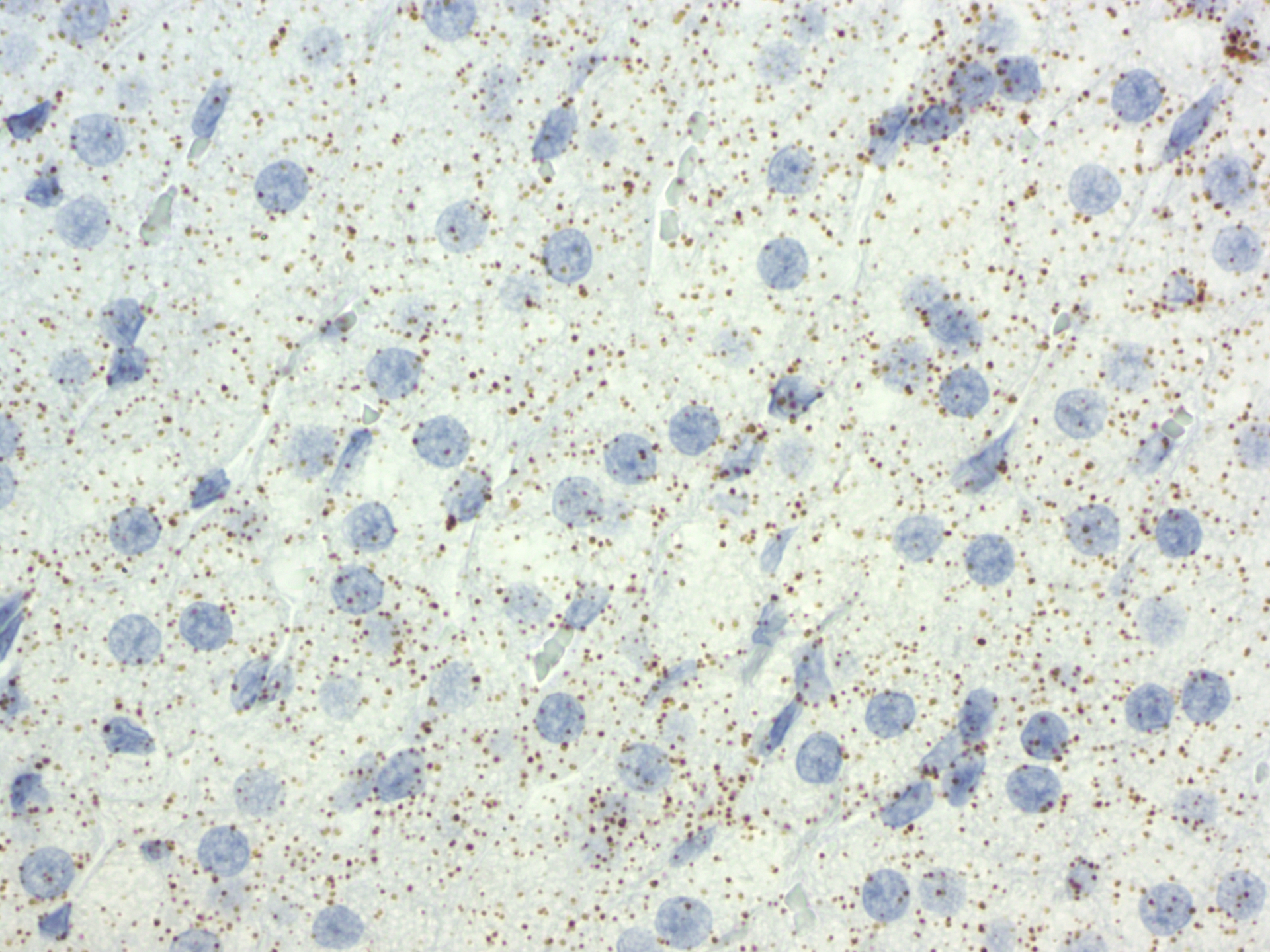

Supplement: Supplementary file 1 [file viruses-10-00280-s001.zip › viruses-297950-SI-1.pdf]

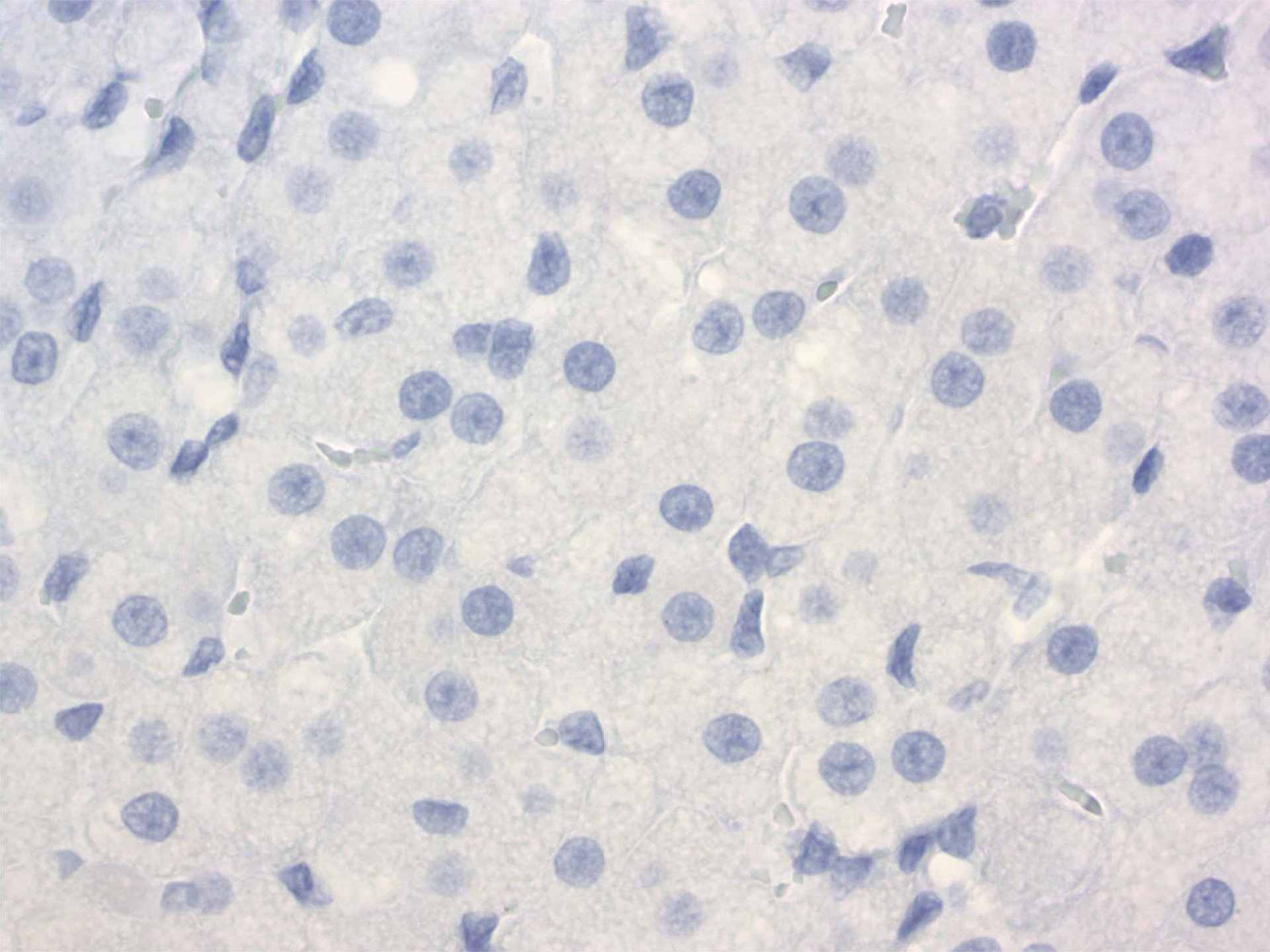

Supplement: Supplementary file 1 [file viruses-10-00280-s001.zip › viruses-297950-SI-2.pdf]

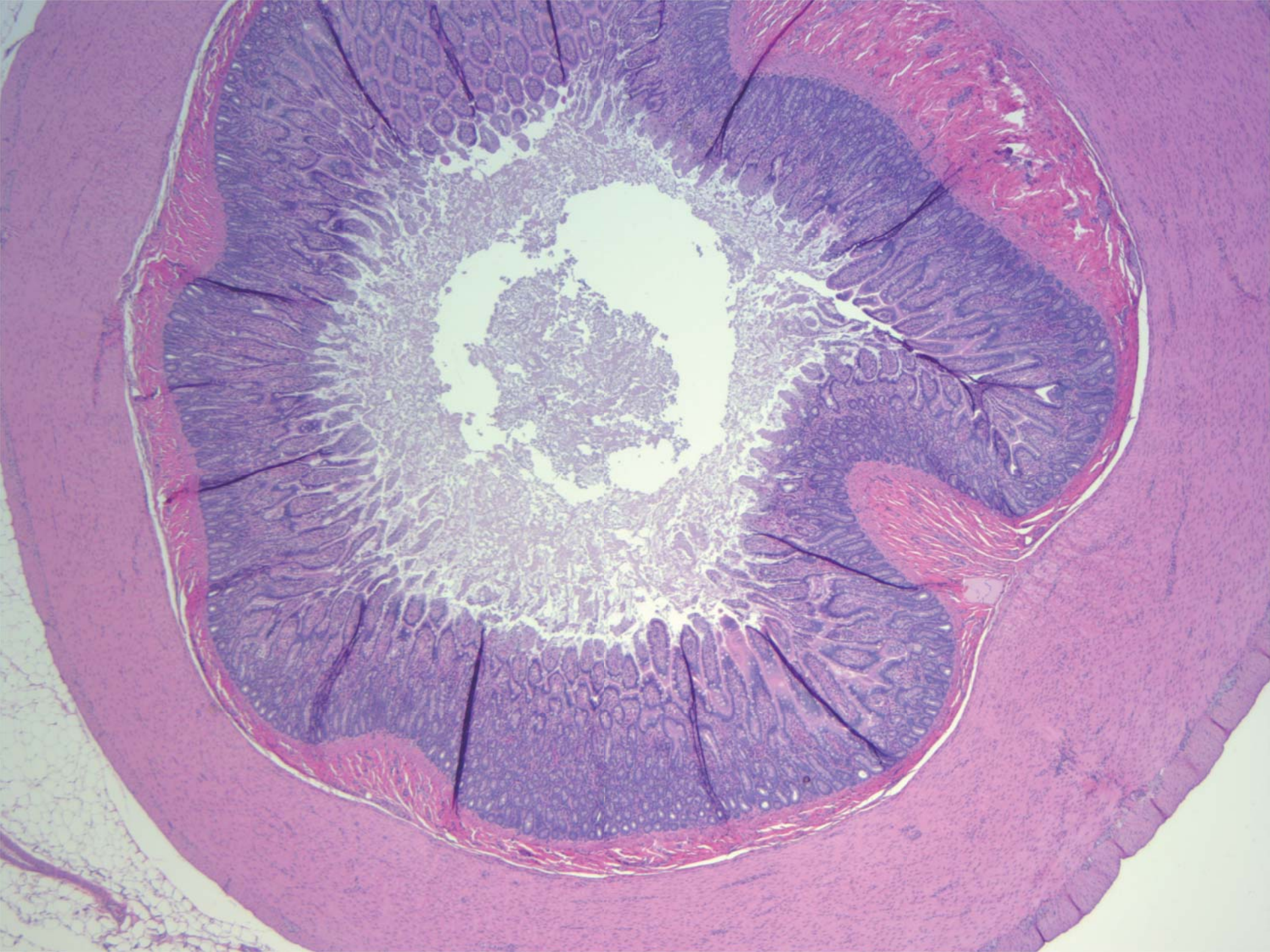

Supplement: Supplementary file 1 [file viruses-10-00280-s001.zip › viruses-297950-SI-3.pdf]

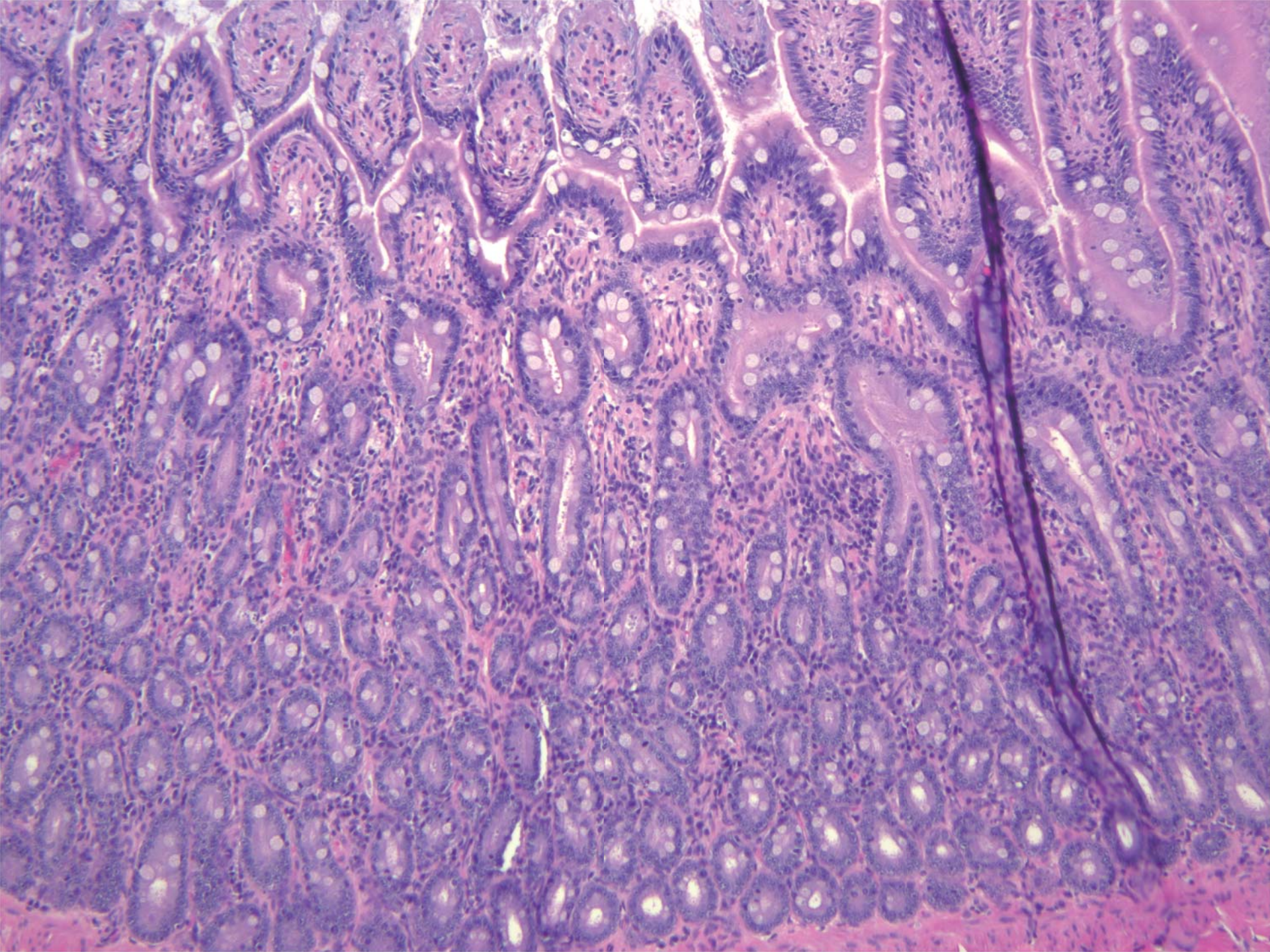

Supplement: Supplementary file 1 [file viruses-10-00280-s001.zip › viruses-297950-SI-4.pdf]
